# Melting-free integrated photonic memory with layered polymorphs

**DOI:** 10.1515/nanoph-2023-0725

**Published:** 2024-01-31

**Authors:** Kaleem Ullah, Qiu Li, Tiantian Li, Tingyi Gu

**Affiliations:** Department of Electrical and Computer Engineering, University of Delaware, Newark, DE 19716, USA; Tianjin Key Laboratory of High-Speed Cutting and Precision Machining, Tianjin University of Technology and Education, Tianjin 300222, China; School of Electronic Engineering, Xi’an University of Posts and Telecommunications, Xi’an, China

**Keywords:** nonvolatile memories, phase transition, layered polymorphs, integrated photonics

## Abstract

Chalcogenide-based nonvolatile phase change materials (PCMs) have a long history of usage, from bulk disk memory to all-optic neuromorphic computing circuits. Being able to perform uniform phase transitions over a subwavelength scale makes PCMs particularly suitable for photonic applications. For switching between nonvolatile states, the conventional chalcogenide phase change materials are brought to a melting temperature to break the covalent bonds. The cooling rate determines the final state. Reversible polymorphic layered materials provide an alternative atomic transition mechanism for low-energy electronic (small domain size) and photonic nonvolatile memories (which require a large effective tuning area). The small energy barrier of breaking van der Waals force facilitates low energy, fast-reset, and melting-free phase transitions, which reduces the chance of element segregation-associated device failure. The search for such material families starts with polymorphic In_2_Se_3_, which has two layered structures that are topologically similar and stable at room temperature. In this perspective, we first review the history of different memory schemes, compare the thermal dynamics of phase transitions in amorphous-crystalline and In_2_Se_3_, detail the device implementations for all-optical memory, and discuss the challenges and opportunities associated with polymorphic memory.

## 1 The history of nonvolatile electronic and photonic memory

**Figure 1:**
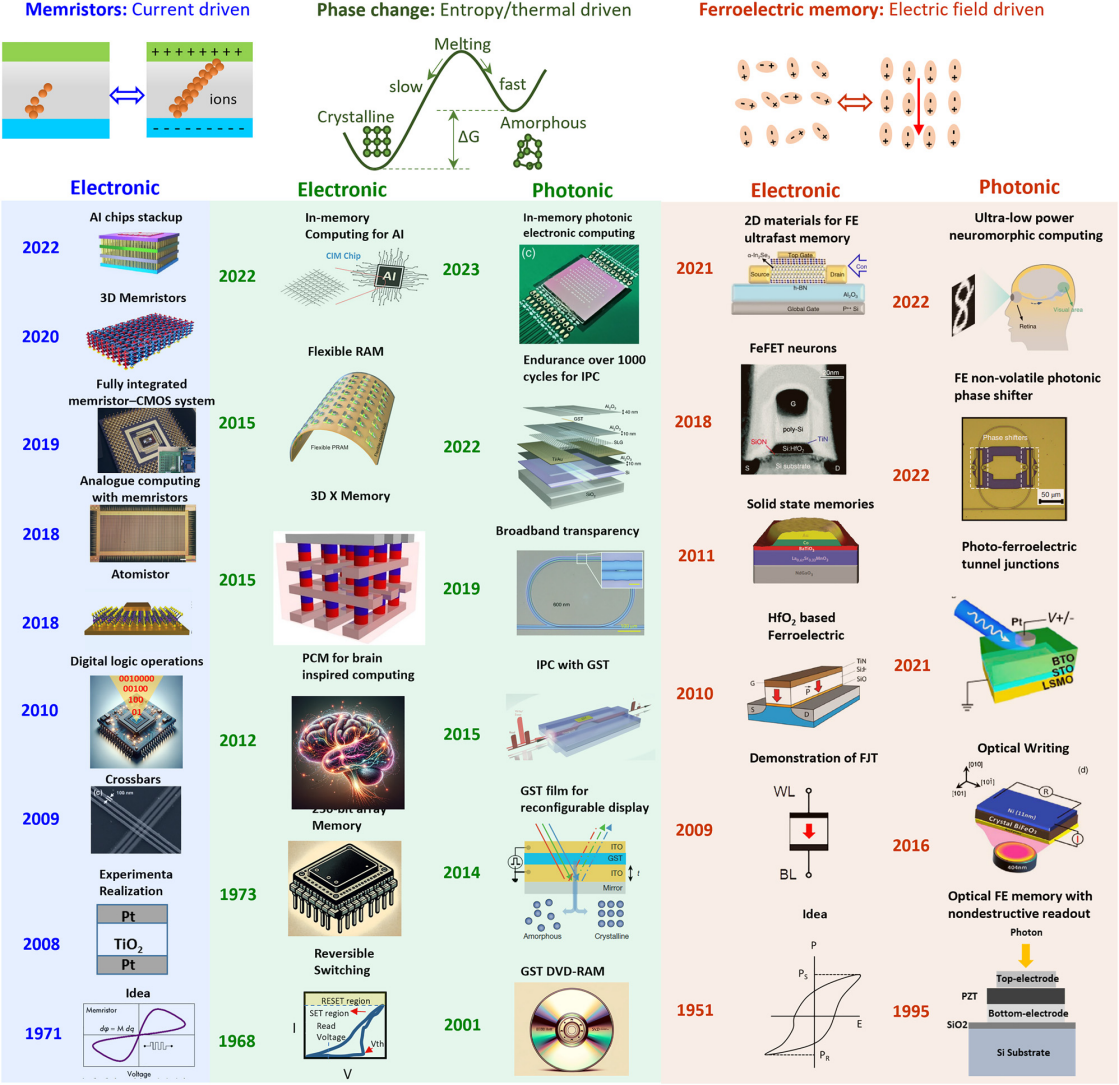
Advancements in semiconductor nonvolatile memory technologies (magnetic types not included). Memristors, PCM (electronic), PCM (photonic), FEM (electronic), and FEM (photonic). The figure is reproduced with the permission of: ref. [3] Copyright 2009, Copyright 2016, American Chemical Society. Ref. [5] Copyright 2018, American Chemical Society. Ref. [6] Copyright 2018, Nature Publishing Group. Ref. [7] Copyright 2019, Nature Publishing Group. Ref. [8] Copyright 2020, Nature Publishing Group. Ref. [10] Copyright 2022, Nature Publishing Group. Ref. [13] Copyright 2015, Nature Publishing Group. Ref. [14] Copyright 2015, American Chemical Society. Ref. [15] Copyright 2022, American Chemical Society. Ref. [17] Copyright 2014, Nature Publishing Group. Ref. [18] Copyright 2015, Nature Publishing Group. Ref. [19] Copyright 2019, Nature Publishing Group. Ref. [20] Copyright 2022, Nature Publishing Group. Ref. [21] Copyright 2023, Nature Publishing Group. Ref. [22] Copyright 2021, AIP Publishing. Ref. [23] Copyright 2021, Nature Publishing Group. Ref. [24] Copyright 2018, The Royal Society of Chemistry. Ref. [25] Copyright 2012, Nature Publishing Group. Ref. [26] Copyright 2022, Nature Publishing Group. Ref. [27] Copyright 2022, Nature Publishing Group. Ref. [28] Copyright 2021, Nature Publishing Group. Ref. [29] Copyright 2016, American Physical Society.

Nonvolatile memories, which retain their device status (resistance or refractive index change) after removing the external drive (such as heat, electric field, current, or illumination), are indispensable components in many stand-alone appliances. Nonvolatile electronic memories have a rich history, and they can be broadly categorized into three primary groups: PCM, memristor, and ferroelectric memory (FEM), operating through thermal-induced atomic restructuring, current-driven ionic dynamics, and electric field-oriented polarization, respectively (as shown in Figure 1) [1], [2], [3], [4], [5], [6], [7], [8], [9], [10], [11], [12], [13], [14], [15], [16], [17], [18], [19], [20], [21], [22], [23], [24], [25], [26], [27], [28], [29], [30]. Other than those main strain memory mechanisms, metal-to-insulator transitions have also been explored in TaS_2_ and VO_2_ [31], [32].

Over the past half-century, all three electronic memory technologies have witnessed significant growth, achieving a high level of maturity in terms of scalability, endurance, and CMOS integration. The pioneering work in electronic memories inspired the development of their photonic counterparts, as both resistance switching and refractive index change originated from atomic structural and/or compositional transformations. The ability to transition between states with large refractive index contrast across large spatial regions makes PCM particularly well-suited for photonic applications. Through material engineering, the optical transparency of chalcogenide O-PCM has been significantly improved for integrated photonic phase memory.

## 2 Motivation and challenges for melting-free photonic memory

**Figure 2:**
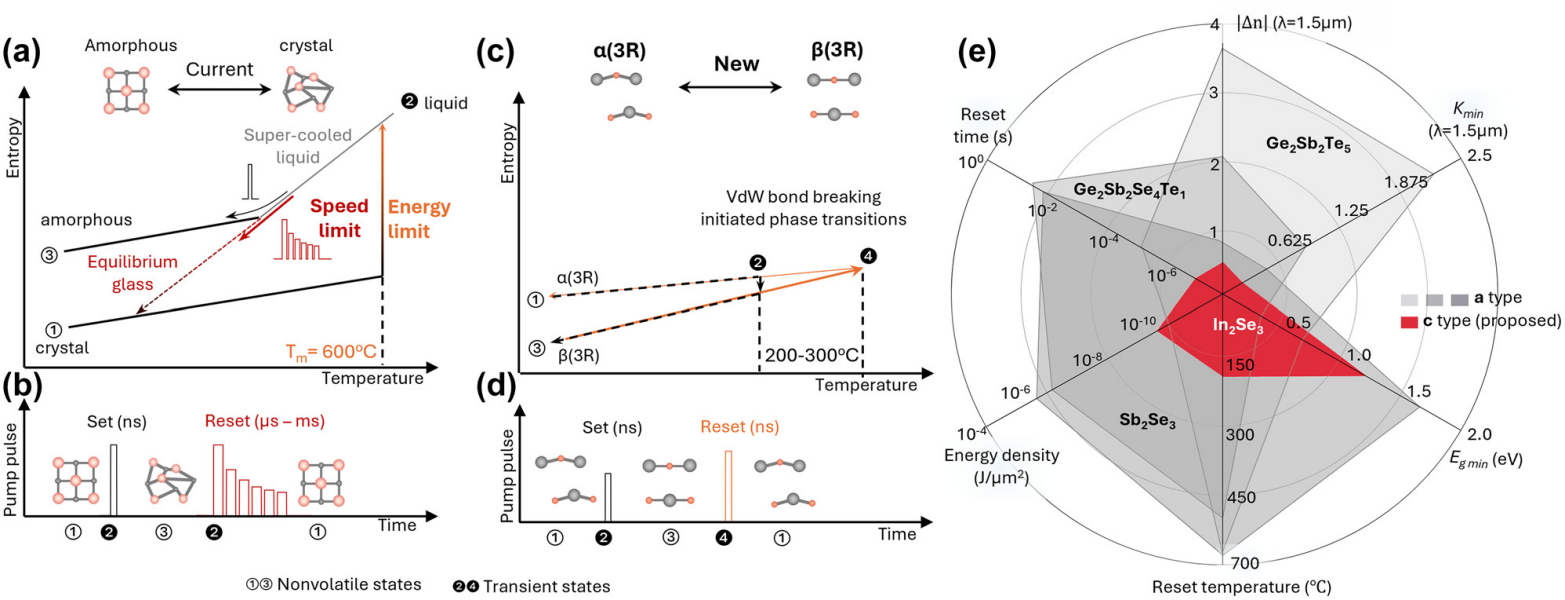
Comparison between amorphous-crystalline phase transitions and polymorphic phase transitions. (a) Thermodynamics of the transition between crystalline and amorphous states in current O-PCMs. The melt–recrystallization requires the excitation energy to break the strong covalent bonds in the amorphous state and slow cooling, allowing the equilibrium glass to transition to ordered crystal structures (top inset). (b) Device SET/RESET dynamics under photothermal or Joule heating pulses. (c) Thermal dynamics of the phase transitions between layered In_2_Se_3_ with the same crystal symmetry. Top inset: atomic picture of convertible layered states (detailed in Figure 3a). (d) Correspondent device response (specified in Section 3). (e) Performance matrix comparison among the PCMs switched between crystalline and amorphous states (gray, following panel a and b) [19], [34], [35], [36], [37], [38], [39], [40], [41], [42], [43], [44] and a polymorphic layered In_2_Se_3_ (red, following c and d) [47]. The refractive index for the higher index state is around 3.7 at 1550 nm.

Chalcogenide compounds and alloys are widely adopted in-memory technologies [33], [34], [35], [36], [37], [38], [39] (Figure 2a and b). Recent research has been focusing on improving optical transparencies by engineering the composition and stoichiometry of compounds [38], [39], [40]. For data writing/SET/amorphization, the energy absorbed needs to heat the material beyond the melting temperature, followed by rapid cooling (picoseconds) for the melting–quench process [41]. During the data erasing/RESET/crystallization, a critical train of pulses (ns to sub-ms) *slowly cools* the Ge_2_Sb_2_Te_5_ from the melting temperature (>600 °C) and recovers the atomic structure, ordering it back to the crystalline state [42], [43]. The melting–recrystallization process consumes the most operation energy, leading to reduced clock/repetition rates and endurance (elemental segregation) and set thermal diffusion distance limited integration density. Efforts have been devoted to reducing the melting temperature and crystallization time [42], [43], [44]. Recent studies have shown that the atomic superlattice reduces switching noise and voltage drift [45], [46]. By transitioning to layered structures and eliminating the use of alloys with random atomic networks, we can unlock the full potential of chalcogenide-based memory technologies, enabling efficient, high-performance, and low-noise photonic memory applications in various fields.

The melting-free nonvolatile phase transition has been demonstrated in a few polymorphic PCMs, such as the transitions between hexagonal and monoclinic crystalline states in monolayer MoTe_2_ [48], [49], [50], [51], and the Joule heating-induced reversible phase change in polymorphic In_2_Se_3_ [52]. The small entropy difference between convertible layered In_2_Se_3_ allows the low-temperature phase transitions in both directions (Figure 2c and d). Both the SET and RESET processes are fast (a few ns) and promise better endurance by occurring at low temperatures (less than 300 °C). Currently, the challenges of polymorphic memory include (1) missing understanding of control factors for phase transition pathways among polymorphs. Given the same stoichiometry, different phase transition pathways and temperatures are reported among different groups [53], [54], [55], [56], [57], [58], [59]; (2) limited studies are found on the polymorphic PCM’s endurance, which is likely to be hindered by oxidation or void formation over the electrodes without careful surface/interface engineering.

Other electronic memory mechanisms (memristors and ferroelectrics) are also melting-free. However, those switching concepts cannot be simply applied to photonic devices. Compared to electronic memory, the formation of photonic memories requires an effective index change over the sub-wavelength area. In the memristor, the effective volume of a single ionic channel (nm scale) is too small for introducing sufficient phase shift or absorption toward the optical modes. The observation of photonic memory in the ferroelectric polymer was reported in 1985 [60]. A few photonic memory devices based on ferroelectric materials have been reported in tunnel junctions [28] and integrated photonic devices [26].

## 3 Structural transition between layered In_2_Se_3_ for single ns pulse RESET memory

The first step of material selection is essential in the way that it might set the upper limit of the device’s performance. Materials composed of fewer elements reduce the chance of element segregation and other parasitic processes [61]. This proposal will focus on the binary compound In_2_Se_3_ and the element Te. Experimental results show that In_2_Se_3_ can be reversibly switched between layered polytypes.

**Figure 3:**
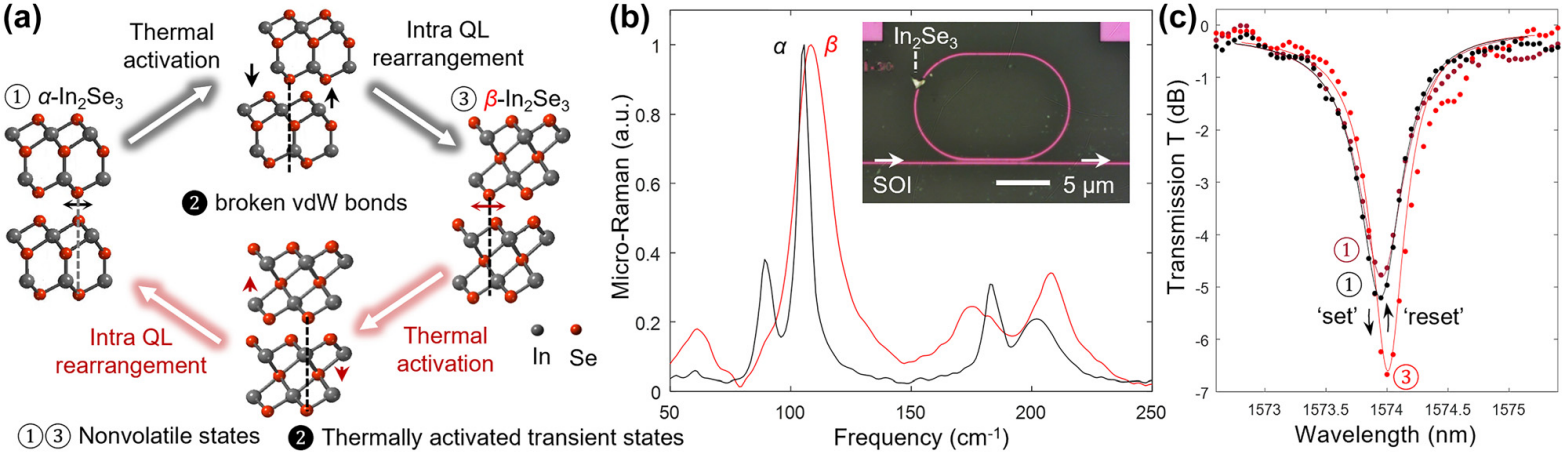
Nonvolatile switching between layered In_2_Se_3_ polytypes. (a) Atomic structure of α- (left) and β-In_2_Se_3_ (right) at room temperature, and after thermally activated shear-glide (top and bottom). The forward and reverse transitions are marked in black and red arrows, respectively. (b) Micro-Raman spectroscopy probed the local state for transferred In_2_Se_3_ flake. Inset: Optical microscope image of a hybrid microring resonator (MRR) made of silicon waveguides. (c) Normalized transmission spectra for the device in panel (b) inset, with α- (black), β*-* (red), and retrieved α-state (dark red) In_2_Se_3_ [47]. The dots are experimental data (with single ns pulse for set and reset). The effective index change is extracted by coupled mode theory fittings (curves).

The PI’s previous work explicated the atomistic pictures for the phase transitions between the layered structures of In_2_Se_3_ [47]. In the *α*-state, the outer Se-atoms between quadruple layers (QLs) are aligned, whereas in the *β*-state, they are located at the interstitial sites of the Se-atoms in the neighboring layers. The structural transition can be initiated by an “interlayer shear glide,” where each QL layer is structurally the same, but the layers are displaced with respect to each other [62]. The QL–QL shear gliding is facilitated when the thermal activation energy exceeds the vdW bond energy, followed by inter-QL distance compression (from α- to β-states) as the outer Se falls into the interstitial sites, or inter-QL distance expansion (from β- to α-states) in the reverse process (Figure 3a).

Our results demonstrate the feasibility of nonvolatile and all-optical switching in the hybrid Si MRR (Figure 3b and c). Both SET and RESET are achieved by a single nanosecond pulse, with higher peak intensities for RESET (illustrated in Figure 2d). Micro-Raman spectra identified the nonvolatile transition between the layered structures of the transferred In_2_Se_3_ flake on silicon MRR (Figure 3b). The effective index change in the small flake In_2_Se_3_ can be derived from the hybrid MRR’s transmission spectra (inset of Figure 3b). The resonance wavelength red-shifted 100 pm after phase transition, and the extinction ratio (ER) increased from 4.45 to 6.27 dB (black and red in Figure 3c). Although both states are transparent at the telecommunication wavelength, the change in ER indicates the optical propagation loss difference.

## 4 Interplay with strain

**Figure 4:**
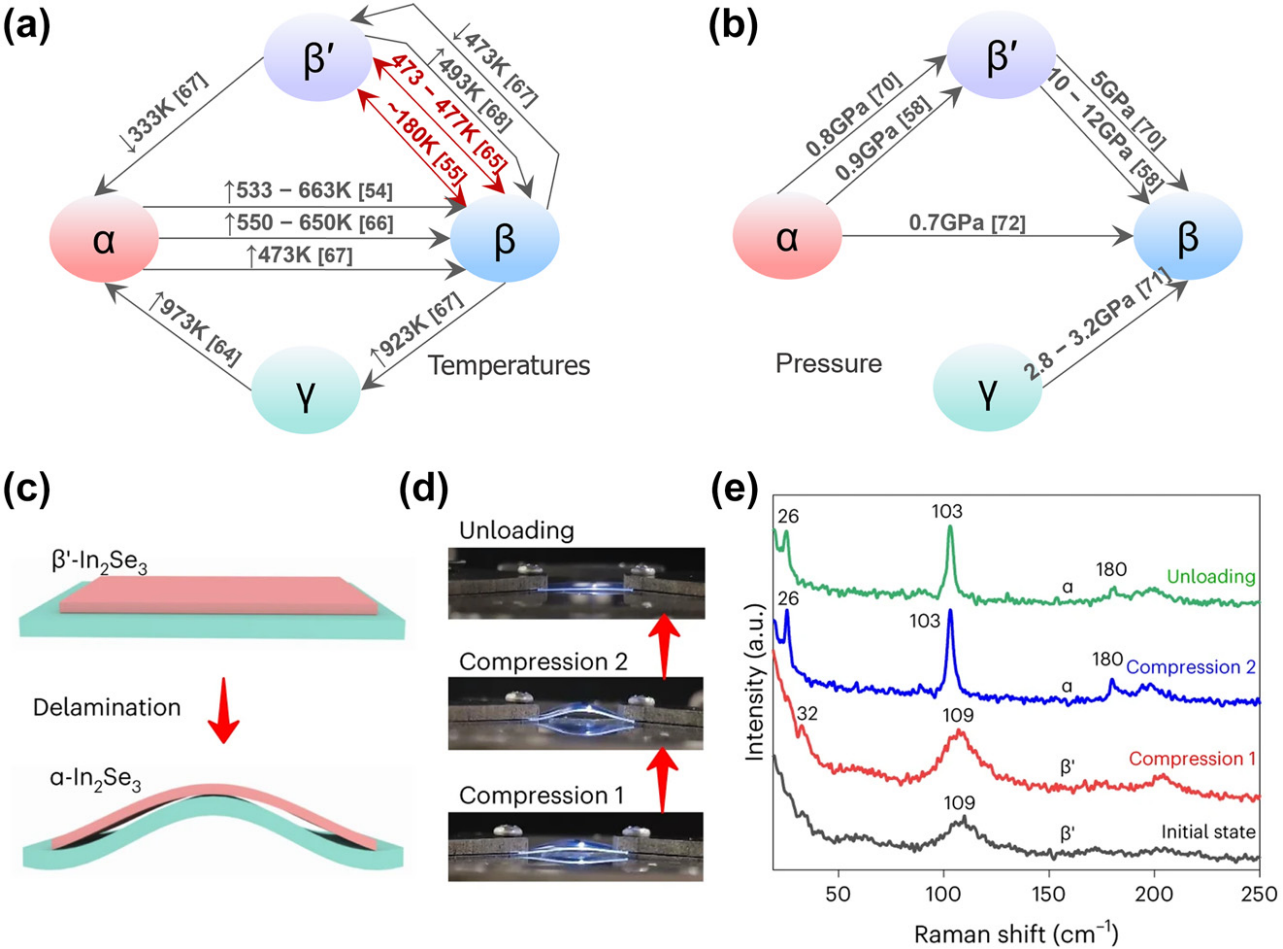
Induced phase transitions of In_2_Se_3_. (a) Temperature-induced phase transition of In_2_Se_3_. The marked temperature is the transformation temperature. The red arrows indicate reversible phase transitions, and the gray arrows indicate irreversible phase transitions or the reversibility of the phase transition has not been tested. ↑ and ↓ donate phase transitions during the heating and cooling processes, respectively. (b) Pressure-induced phase transitions of In_2_Se_3_. (c–e) Strain-induced phase transitions of In_2_Se_3_. From left to right are the schematic illustration of the strain stage, serial photos of the straining device during the compression process, and the *in situ* Raman spectra of β′-In_2_Se_3_ films in different compression states. (c–e) The figure is reproduced with the permission of ref. [73] Copyright 2023, Nature Publishing Group.

As a polymorphic material with multiple crystalline states, the phase transmission dynamics and pathway are one of the most intriguing challenges. Temperature-induced In_2_Se_3_ phase transition pathways with different initial and final states have been reported at a set of temperatures, some of which are reversible and nonvolatile [54], [55], [63], [64], [65], [66], [67], [68] (Figure 4a). At the device level, electrically driven reversible phase transitions have been achieved with Joule heating, including the transition between β and γ phases [52], and the transitions from α to β′ and α to γ [69].

Independently, pressure can induce phase transitions in In_2_Se_3_, which were considered irreversible [58], [70], [71], [72] (Figure 4b). The latest research reveals that such phase transitions in layered In_2_Se_3_ can be reversible. By unloading residual tensile strain in the growth state of β′-In_2_Se_3_ using nanomechanical hands, it can be transformed into α-In_2_Se_3_. Conversely, applying stretching to α-In_2_Se_3_ on a TEM grid via copper foil enabled its reversion back to β′-In_2_Se_3_ [69]. Interestingly, residual strain in the growth state of layered β′-In_2_Se_3_ thin films can also be released when transferring the film to a flexible substrate and subjected to repeated bending. Using this method of unloading strain-induced phase transition, large-area layered α-In_2_Se_3_ thin films have been obtained (Figure 4c–e) [73].

In a noteworthy breakthrough, reversible thermally driven phase transitions between the β′ and α phases of layered In_2_Se_3_ have been actualized, aided by localized strain at surface wrinkles and ripples. On this premise, phase-modulating devices have been realized with minimal light insertion loss (less than 0.012 dB/μm) [59]. This research unfolds strain-induced phase transitions that may have to be considered in the fabrication process of nonstrain-driven phase-change memory devices, such as thin-film transfers.

## 5 Other layered polymorphs for electronic and photonic memories

Chalcogenides with layered structures are the most prominent examples of two-dimensional materials that exhibit polymorphic phase transitions at temperatures that are nearly equal to ambient temperature [74]. Chalcogen elements such as sulfur (S), selenium (Se), and tellurium (Te) possess comparatively lower electronegativity values when contrasted with oxygen and the majority of halogen elements (fluorine, chlorine, bromine) [75]. Consequently, in a substantial number of these chalcogenides, there is a competitive interaction between ionic and covalent bonds [76]. This competition gives rise to structural polymorphs that have various bonding arrangements with energy levels that are similar. As a result, even minor external influences can trigger these polymorphic transitions within these substances. Recent studies have shed light on the fascinating polymorphic phase transitions in two-dimensional materials, particularly those derived from chalcogenides with layered structures [77], [78], [79]. These materials, including group VI transition-metal dichalcogenide monolayers (MX_2_, where M = Mo or W and X = S, Se, or Te), exhibit a unique interplay between ionic and covalent bonding, leading to various structural polymorphs such as 2H, 1T, and 1T′ phases. These phases have distinct coordination patterns and electronic properties, ranging from semiconducting to metallic and topological insulators. For instance, the 2H phase is known for its trigonal prismatic coordination and possesses an optical bandgap of 1.0–2.5 eV, making it suitable for semiconducting applications [76]. On the other hand, the 1T phase, which experiences Peierls distortion [80], changes into unique 1T′, which may carry the quantum spin Hall effect and superconductivity. The ability of these materials to undergo phase transitions in response to various stimuli such as temperature, strain, and electric fields opens new avenues in photonic applications [81]. This is particularly relevant for materials like MoTe_2_, where transitions between the 2H and 1T′ phases can be induced by nonchemical factors, offering a versatile approach for the development of advanced photonic devices [82]. Theoretical and experimental insights into these polymorphic transitions are needed to evaluate the feasibility of reversible phase transitions [83].

## 6 Applications

In the development and implementation of photonic memory applications, several key factors play a pivotal role in determining their success. The selection of materials stands out as a critical consideration; these materials must exhibit optimal optical properties such as a high refractive index contrast and low optical loss, while remaining compatible with standard fabrication techniques. What sets photonic memory apart from electronic memory is its potential for ultra-high-speed operation, making the speed of data writing, storage, and retrieval a crucial aspect. Another important facet is the seamless integration of photonic memory with existing electronic systems, which necessitates efficient interfacing and signal conversion between electronic and optical components. Notably, photonic systems are renowned for their energy efficiency, which proves advantageous in minimizing power consumption for data storage and retrieval – a critical factor for large-scale applications. Furthermore, scalability is paramount, requiring the technology to support higher data densities and compact integration, essential for widespread adoption across various applications, from consumer electronics to large data centers. Ensuring data retention and stability are vital, with photonic memory devices expected to preserve data integrity over extended periods and under various environmental conditions. Cost-effectiveness is another significant factor that encompasses not only initial manufacturing expenses but also long-term operational costs, ensuring commercial viability in comparison to existing technologies. Additionally, wavelength multiplexing capabilities, enabling increased data capacity through multiple wavelengths, represent a crucial feature of photonic memory systems. Lastly, like all memory systems, photonic memory necessitates robust error correction mechanisms to maintain data integrity, a necessity that becomes increasingly important as the technology scales. Each of these elements collectively influences the performance and feasibility of photonic memory systems. The relative importance of these factors may vary based on specific applications and existing technological limitations. For a succinct summary, refer to Table 1, which provides a comparative analysis of photonic memory and traditional memory technologies, focusing on the aspects discussed above.

**Table 1:** Comparative overview of memory technologies.

	Photonic memory	Electronic memory (e.g., DRAM)	Magnetic memory (e.g., HDD)
Speed	Ultra-high-speed data transfer	High-speed data transfer	Slower than electronic
Energy efficiency	High (low power consumption)	Moderate	Low (higher power usage)
Scalability	High potential for miniaturization and integration	Limited by electronic constraints	High density possible but limited scalability
Data retention	Long-term stability potential	Volatile (requires power)	Nonvolatile
Cost	Currently high, potential for reduction	Moderate, well-established manufacturing processes	Generally lower cost
Error correction	Developing needs more research	Advanced error correction techniques	Advanced error correction techniques

## 7 Conclusion and perspective

In conclusion, this perspective highlights the pivotal role that layered polymorphic materials can play in the evolution of integrated photonic memories. The small and collective atomic displacements during switching in these layered structures suggest a promising avenue for improving material fatigue and extending device life cycles. The use of layered chalcogenide In_2_Se_3_ is emphasized as a strong substitute for conventional melt–recrystallization methods. Its low entropic phase transitions between layered structures are particularly noteworthy for their ability to address the energy efficiency and speed limitations that affect current integrated photonic memory devices. The transition from the β-state to the α-state of In_2_Se_3_ can be easily achieved by applying a nanosecond pulse, as depicted in Figure 2. This rapid phase change is notably faster than the microsecond-to-millisecond transitions commonly seen in amorphous crystallization processes. In addition, the optical transparency of In_2_Se_3_ in both states at telecommunication wavelengths ensures low insertion loss, making it a strong candidate for phase-only memory devices. Such phase change properties place polymorphic layered materials, and especially In_2_Se_3_, at the forefront of future research for more energy-efficient, fast, and durable photonic memory solutions.

The refractive index change in layered In_2_Se_3_ is only 1/2 of GSST, but its figure of merit (FoM) as optical PCM is among the top of all the optical PCMs, which is attributed to the low absorption and wide optical bandgap for both states. The definition of FoM = Δ*n*/Δ*k* is provided by the ref. [19]. Δ*n* is the refractive index difference and Δ*k* is the extinction ratio contrast (associated with absorption) between the initial and final states. The refractive index contrast between two states is directly associated with their atomic structural difference. The topological similarities between the crystalline phase change materials bring advantages of ultrafast switching and low phase change temperature/energy. The same atomic structural features lead to a low refractive index difference Δ*n* = 0.45. Both states (α- to β-In_2_Se_3_) are transparent in the near-infrared (1550 nm). Theoretically (from ab-initial calculation), the absorption (parameterized by the extinction coefficient *k*) of both states is zero (Δ*k* = 0) at 1550 nm wavelength. Experimentally, due to the surface roughness and defect absorptions, we measured the Δ*k* of 0.02 (*k*_α-In2Se3_ = 0.02, *k*_β-In2Se3_ = 0.04). The experimentally measured In_2_Se_3_ FoM = 25 at 1550 nm, and the theoretical FoM for In_2_Se_3_ is infinite.

Here are the challenges and opportunities for the future development of the layered polymorphs:

**Contact Engineering:** Contact engineering is a universal challenge for introducing all new semiconductor materials for electronic and optoelectronic device integrations [84], [85]. The application of substitutional doping techniques has been found effective in reducing contact resistance in bulk semiconductors. The effect of such an approach on layered polymorphic materials may be modified by the parasitic strain [84], [86], [87]. The progress made in two-dimensional material-based transistors may help in reducing contact resistance associated with memory failure: (1) semi-metallic bismuth has been shown to reduce contact resistance to approximately 123 Ω μm [88]; (2) van der Waals gaps have proven to be effective in the creation of contacts that are free from interactions and defects [89]; and (3) graphene-assisted metal transfer printing methods have demonstrated almost a 100 % yield in transferring metal electrodes, providing a versatile strategy that could be adapted for polymorphic materials [90].

**Elemental Doping:** Elemental doping has trailed for conventional PCMs [91]. One established method to enhance the structural stability of amorphous PCMs involves tuning the composition of GST alloys away from the GeTe–Sb_2_Te_3_ pseudo-binary line [92], [93]. Furthermore, Sb-rich PCMs have been identified for their more stable amorphous phases, substantiating the role of elemental doping in achieving greater structural stability [94], [95], [96], [97], [98], [99]. Notably, changes in quenching rates and the alloying of Ge into amorphous Sb have been shown to increase the rigidity of chemical bonds, thereby hindering crystallization kinetics and stabilizing the amorphous state [94]. Low-concentration dopants like Ag and In have also been effective in increasing the viscosity near room temperature, further stabilizing the amorphous phases [85]. These doping methods aim to prolong the stability of the amorphous phase, improving data retention and overall device performance [96], [97], [98], [99]. For the crystalline material In_2_Se_3_, elemental doping might be challenging as it needs to be introduced during epitaxial growth. If successful, the right doping might help reduce the parasitic strain effect in the phase transition process.

**Leveraging the Strain effect**: While it’s known that strain can trigger phase changes in this material (Section 4), applying it in a controlled manner is not straightforward. Various factors during the fabrication process, such as temperature changes, can introduce unexpected levels of strain [73]. This makes it difficult to predict the material’s behavior in practical applications. In addition, the strain must be applied uniformly to obtain consistent results, which adds a layer of complexity. The interplay between element doping (intended or parasitic) and strain might result in unpredictable phase transition pathways in polymorphic PCM, which have multiple convertible crystalline states.

**Material Preparation**: The molecular beam epitaxy demonstrates large-area growth single phase and single crystalline In_2_Se_3_ films [100]. Chemical Vapor Deposition (CVD) techniques result in simultaneous occurrence of numerous phases [101], [102]. Direct synthesis of pure phase β′ films have not been achieved by current, and they are exclusively seen in mixed phases [101], [103]. CVD yields crystalline α-phase In_2_Se_3_ flakes and is feasible for growing at a large scale [104], [105]. Additionally, the concentration control of precursors in traditional CVD, especially over long source-to-substrate transport distances, remains a technical hurdle that needs to be overcome [73]. Therefore, advancements in synthesis techniques are essential for maximizing the capabilities of In_2_Se_3_ in nonvolatile memory applications.

**Improvement of Endurance**: Oxidation (which can be accelerated with laser heating/overdose) is considered the primary mechanism for chalcogenide degradation. An airtight cladding layer can block the chalcogenide’s interaction with the oxygen in the air [106]. The proper laser dosage reduces the chance/opportunity of thermally induced atomic structural deformation. An optimized thin passivation layer such as ITO or Al_2_O_3_ would be good for the cyclability of the devices. Reduced peak power and shorter excitation wavelength can be improved to reduce the chemical change of the material.
